# Polymorphisms in enterovirus 71 receptors associated with susceptibility and clinical severity

**DOI:** 10.1371/journal.pone.0206769

**Published:** 2018-11-05

**Authors:** Ting-Yu Yen, Wei-Liang Shih, Yi-Chuan Huang, Jian-Te Lee, Li-Min Huang, Luan-Yin Chang

**Affiliations:** 1 Department of Pediatrics, China Medical University Children’s Hospital, China Medical University, Taichung, Taiwan; 2 Institute of Epidemiology and Preventive Medicine, College of Public Health, National Taiwan University, Taipei, Taiwan; 3 Department of Pediatrics, Kaohsiung Chang Gung Memorial Hospital and Chang Gung University College of Medicine, Kaohsiung, Taiwan; 4 Department of Pediatrics, National Taiwan University Hospital, Yun-Lin Branch, Yunlin, Taiwan; 5 Department of Pediatrics, National Taiwan University Hospital, College of Medicine, National Taiwan University, Taipei, Taiwan; University of Hong Kong, HONG KONG

## Abstract

**Objective:**

To evaluate the association of enterovirus 71 (EV71) susceptibility and clinical severity with polymorphisms in EV71 receptors, including human scavenger receptor class B member 2 (SCARB2), P-selectin glycoprotein ligand-1 (PSGL-1), and annexin II (ANXA2).

**Methods:**

We enrolled laboratory-confirmed EV71 cases and healthy age- and gender-matched controls in Taiwan from 2000 to 2012. We detected genetic polymorphisms in SCARB2, PSGL-1, and ANXA2 and correlated the results with EV71 susceptibility and severity.

**Results:**

We collected 599 EV71 cases and 98 controls. Among EV71 patients, the male to female ratio was 1.61, and the mean age was 2.99±2.47 years. For clinical severity, 117 (19.6%) had severe central nervous system involvement with or without cardiopulmonary failure. For outcomes, 46 (7.7%) had sequelae, and 14 (2.3%) died. SCARB2 polymorphisms (rs6824953 and rs11097262) were associated with susceptibility to EV71 infection (OR 1.60, 95% CI 1.07–2.39; and OR 1.64, 95% CI 1.09–2.47, respectively). PSGL-1 polymorphisms (rs7137098 and rs8179137) were significantly associated with severe EV71 infection (OR 1.46, 95% CI 1.1–1.96; and OR 1.47, 95% CI 1.07–2.03, respectively).

**Conclusions:**

SCARB2 polymorphisms (rs6824953 and rs11097262) might be associated with EV71 susceptibility. PSGL-1 polymorphisms (rs7137098 and rs8179137) were associated with severe EV71 infection.

## Introduction

Enterovirus 71 (EV71) belongs to human enterovirus species A of the family *Picornaviridae* and has been regarded as the most important neurotropic enterovirus after the near eradication of the polioviruses by vaccine. Since its discovery in 1969, EV71 outbreaks have been reported periodically throughout the world, especially in Southeast Asia over the past decades [1]. In Taiwan, EV71 has become one of the most important endemic diseases in children with an interval of three to five years after the first documented large outbreak in 1998 [2–6]. Patients with EV71 infection display very diverse clinical symptoms, ranging from asymptomatic or mild hand-foot-and-mouth disease (HFMD) and herpangina (HA) to severe neurological and cardiopulmonary disease, and even fatal disease [7–10]. Whether this is related to host, social, behavioral or environmental factors remains unanswered. It is very important to delineate host susceptible genes that contribute to EV71 infection, which would assist in predicting individual and population risk, as well as help clarify the pathogenesis and provide further therapeutic strategies for EV71 infection.

Individual susceptibility to infectious disease seems to be variable. Tumor necrosis factor α (TNF-α) promoter polymorphism is related to higher mortality rates and morbidity rates of sepsis [11]. In addition, allelic variants have been associated with increased or decreased susceptibility to human immunodeficiency virus, hepatitis B, meningococcus, tuberculosis and so on [12,13]. We have also demonstrated pronounced elevations in inflammatory cytokines such as TNF-α, interleukin 1β (IL-1β), and IL-6 in fatal EV71 cases [14, 15]. In addition, HLA-A33 was found to be associated with EV71 susceptibility in Taiwanese patients [16]. Zhang et al. proved that gene polymorphisms in myxovirus resistance A, an antiviral protein induced by type I interferons α and β (IFN-α and IFN-β) that can inhibit virus replication, are associated with susceptibility to EV71 infection in a Chinese population [17]. Recently, several gene polymorphisms in cytokines and chemokines, such as IFN-γ, IL-8, IL-10, IL-17F, C-C motif chemokine ligand 2, and C-X-C motif chemokine 10, have been demonstrated to be associated with susceptibility to EV71 infection in Chinese patients [18–21]. All this evidence suggests that host genetic factors may play an important role in susceptibility to EV71 infection and its clinical severity.

Viral receptors have an essential role in the early steps of viral infection and are a primary determinant of host range and tissue tropism. Recently, two human transmembrane proteins, scavenger receptor class B, member 2 (SCARB2, also known as lysosomal integral membrane protein II or CD36b like-2) [22] and P-selectin glycoprotein ligand-1 (PSGL-1) [23], were identified as functional receptors for EV71. SCARB2 is a type III double-transmembrane protein with N- and C-terminal cytoplasmic tails and is located primarily in lysosomes and endosomes [24, 25]. SCARB2 is expressed ubiquitously in human tissues [26]; therefore, it might be involved in systemic EV71 infections [22]. In contrast, P-selectin glycoprotein ligand-1 (PSGL-1; CD162), a sialomucin membrane protein expressed on leukocytes, has a major role in early stages of inflammation [27, 28] and is expressed on dendritic cells of lymph nodes and macrophages in the intestinal mucosa [27], which is thought to be the primary site of EV71 entry and replication. Stable PSGL-1 expression allows EV71 entry and replication and the development of cytopathic effects [23]. In addition, annexin II (ANXA2), a member of the annexin family on the surface of endothelial cells, was found to be a cellular factor involved in the early stages of EV71 infection [29]. The interaction was specific to EV71, and the binding of EV71 to ANXA2 on the cell surface enhanced viral entry and infectivity, especially at a low infective dose [29]. Several lines of evidence suggest that cellular receptors and adhesion molecules play critical roles in efficient EV71 infection and the development of disease in humans, but the associations of these genetic polymorphisms have rarely been investigated clinically. In 2014, Cai et al. tested four single-nucleotide polymorphisms (SNPs) [2'-5'-oligoadenylate synthetase 1 (OAS1) rs10774671, PSGL-1 rs2228315, SCARB2 rs41284767 and IL28B rs12979860] in 333 HFMD samples and 163 control samples [30]. They demonstrated that the OAS1 rs10774671 SNP GG genotype contributed to coxsackievirus A16 susceptibility and was associated with the development of mild HFMD. However, there has been no study investigating genetic polymorphisms in SCARB2, PSGL-1, and ANXA2 focused on children with EV71 infections until now. In this study, we evaluated genetic polymorphisms in EV71 receptors (SCARB2, PSGL-1) and the adhesion molecule (ANXA2) and correlated the results with EV71 susceptibility and clinical severity.

## Materials and methods

### Case enrollment

We conducted a multi-institutional study and enrolled children in six hospitals (three tertiary and three regional hospitals) located in three major areas (Taipei in the northern area, Taichung in the western area and Kaohsiung in the southern area) in Taiwan. The Institutional Review Boards of National Taiwan University Hospital approved this study with approval number 201002049R.

Written informed consent was obtained from the patients’ parents or guardians. Clinically, patients had HFMD, herpangina or febrile illness and laboratory-confirmed EV71 infection, which was based on the positive viral isolation of EV71, positive EV71 VP1 genotyping, or a 4-fold increase in EV71 neutralizing antibody serotiter between a serum sample taken at the acute stage of infection and that taken at the convalescent stage. Their clinical data, including demographic data, clinical manifestations, laboratory data, diagnosis, treatment and clinical outcomes, were recorded. Clinical severity was divided into two groups: the mild group had uncomplicated HFMD/HA, febrile illness or mild central nervous system (CNS) involvement being myoclonic jerk or aseptic meningitis, whereas the severe group had severe CNS involvement such as encephalitis, polio-like syndrome or encephalomyelitis or severe CNS involvement combined with or without cardiopulmonary failure. Cases of aseptic meningitis involved headaches, irritability and cerebrospinal fluid (CSF) pleocytosis (>5x10^6^ leukocytes/L) but no altered level of consciousness or focal signs. Cases of encephalitis had an altered level of consciousness plus CSF pleocytosis; cases of poliomyelitis-like syndrome had acute limb weakness and decreased reflex and muscle strength; and cases of encephalomyelitis had both encephalitis and poliomyelitis-like syndrome. Patients with cardiopulmonary failure after severe CNS involvement were those who had experienced cardiopulmonary failure 2–36 hours (median 12 hours) after manifestations of EV71 CNS infection; these children all required inotropic agents, endotracheal intubation and ventilator support, and they had cardiopulmonary failure due to medullary damage without evidence of independent pneumonia, myocarditis, or bacterial sepsis. Clinical outcomes included complete recovery, with sequel or death.

For the control group, age- and sex-matched healthy children were selected. The participants in the control group were recruited in Chang Gung Memorial Hospital in Taoyuan, and informed consent was obtained from their parents or guardians. They had no history of HFMD or HA, and all of their EV71-neutralizing antibody test results were negative.

### Single nucleotide polymorphism (SNP) selection and genotyping of the SCARB2, PSGL-1 and Annexin II genes

SNP genotype data for the SCARB2, PSGL-1 and Annexin II genes were downloaded from the phase II Nov08 HapMap SNP database and NCBI database, and the data were analyzed with Haploview4.2 software. We selected SNPs for further association study based on the r^2^ linkage disequilibrium with the following criteria: the minor allele frequency (MAF) cutoff was 0.05, and the correlation coefficient (r^2^) threshold was 0.8. S1 Table provides details of all tag SNPs tested in this study. Genomic DNA was extracted from ethylenediaminetetraacetic acid (EDTA)-treated whole blood using the Puregene DNA Isolation Kit (Gentra Systems), in accordance with the manufacturer’s instructions. SNP genotyping was performed by Sequenom MassARRAY platform with iPLEX gold chemistry (Sequenom, San Diego, CA). By following the manufacturer’s guide, the specific PCR primer and extension primer sequences were designed with the Assay Designer software package (v.4.0). A multiplex polymerase chain reaction (Multiplex PCR) assay was performed. Unincorporated dNTPs were deactivated, and the single base extension reaction was performed using an iPLEX enzyme, a terminator mixture, and an extension primer mixture. After purification, the purified primer extension reaction was loaded onto a matrix pad of a SpectroCHIP (Sequenom). SpectroCHIPs were analyzed using a MassARRAY Analyzer 4, and the calling by clustering analysis was done with TYPER 4.0 software.

### Statistical analysis

Hardy-Weinberg equilibrium was tested for each SNP to detect any deviation in the control samples. Allelic and genotypic frequencies of SCARB2, PSGL-1 and Annexin II SNPs were compared between EV71 cases and the control group to identify polymorphisms associated with susceptibility and between the mild group and the severe group to find polymorphisms associated with severe CNS complications by chi-square and Fisher’s exact tests. Odds ratios and 95% confidence intervals were calculated using multiple logistic regression after adjustment for gender and age. Bonferroni’s adjustment and permutation testing with 5000 permutations were used to correct for multiple comparisons. These analyses were performed with SAS software version 9.3 (SAS Institute, Cary, NC), as appropriate. Pairwise linkage disequilibrium index (measured as r^2^) was estimated by using Haploview software, version 4.2 [31].

## Results

### Demographics, clinical severity and outcomes

A total of 599 patients with laboratory-confirmed enterovirus 71 from northern, western, and southern Taiwan and 98 normal control cases were included in the study. Table 1 shows the clinical diagnoses and outcomes of all 599 EV71 cases. As for the demographics, the male to female ratio was 1.62, and the majority were children younger than 5 years (mean: 2.99±2.47).

**Table 1:** Clinical diagnoses and outcomes of 599 EV71 cases.

Clinical characteristics/diagnosis	Number (%) (N = 599)	Clinical Outcome		
		Recovery (N = 539)	Sequelae (N = 46))	Expired (N = 14)
Age				
Mean (SD)	2.99 (±2.47)	3.14 (±2.52)	1.29 (±0.89)	2.84 (±2.11)
Gender				
Male/Female	370/229 (1.62)	331/208 (1.59)	29/17 (1.71)	10/4 (2.50)
Clinical severity				
Mild group^a^	482 (80.5%)	482	0	0
Severe group^b^	117 (19.5%)	57	46	14

EV71, enterovirus 71; SD, standard deviation; CNS, central nervous system

^a^The mild group included uncomplicated cases and cases with myoclonic jerk or aseptic meningitis.

^b^The severe group included severe CNS cases (encephalitis, polio-like syndrome or encephalomyelitis) with or without cardiopulmonary failure.

As for clinical severity, the mild group included 195 (32.6%) uncomplicated EV71 cases and 287 (47.9%) cases of mild CNS involvement, whereas the severe group consisted of 61 (10.2%) severe CNS cases and 56 (9.4%) cases of severe CNS involvement plus cardiopulmonary failure. Most EV71 patients recovered completely, but 46 (7.7%) in the severe group had sequelae. Furthermore, 14 (2.3%) patients with severe CNS involvement plus cardiopulmonary failure had a fatal outcome.

### Association of polymorphisms with EV71 susceptibility

Table 2 shows the genotypic and allelic frequencies of SNPs between the first cohort of 217 EV71 cases collected before 2011 and the normal controls. Table 2 lists only SNPs with p values less than 0.10, and all the detailed results are shown in S2 Table. We found that the genetic polymorphisms (rs6824953, rs6825004 and rs11097262) in EV71 receptor SCARB2 may be significantly associated with the susceptibility to EV71 infection by multiple logistic regression analyses after adjustment for age and sex. For the SCARB2 rs6824953 polymorphism, children with the G allele had increased susceptibility to EV71 infection compared with those with the C allele (OR 1.94, 95% CI 1.19–3.17, P = 0.01). For SCARB2 rs6825004, children with the C allele were more susceptible to EV71 (OR 1.75, 95% CI 1.07–2.87, p = 0.03) than children with the G allele. Similarly, for the SCARB2 rs11097262 polymorphism, children bearing the C allele had increased susceptibility to EV71 infection compared with those with the T allele (OR, 2.07; 95% CI, 1.25–3.44, P = 0.0046). For the SNPs in PSGL-1 (SELPLG) and ANXA2, there was no significant difference between the EV71 case and control groups.

**Table 2:** Susceptible host gene polymorphisms in normal cases and EV71 cases before 2011.

Gene	Genotype/	Normal cases (N = 98)		EV71 cases (N = 217)		Logistic regression analysis		
(SNP)	Allele	No.	%	No.	%	OR^a^	(95% CI)^a^	P value^a^
**SCARB2**								
**rs17001594**	GG	37	37.8	60	27.9	Ref.		
**Intron**	GA	43	43.9	112	52.1	2.06	(0.98, 4.35)	0.06
	AA	18	18.4	43	20.0	1.31	(0.49, 3.51)	0.60
	G	117	59.7	232	54.0	Ref.		
	A	79	40.3	198	46.1	1.25	(0.78, 1.99)	0.35
**rs6824953**	CC	15	15.3	22	10.2	Ref.		
**Intron**	GC	53	54.1	86	40.0	1.23	(0.39, 3.83)	0.72
	GG	30	30.6	107	49.8	3.31	(1.03, 10.64)	0.04
	C	83	42.4	130	30.2	Ref.		
	G	113	57.7	300	69.8	1.94	(1.19, 3.17)	0.01
**rs6825004**	GG	13	13.3	19	8.8	Ref.		
**Intron**	CG	51	52.0	100	46.5	3.42	(0.85, 13.75)	0.08
	CC	34	34.7	96	44.7	5.68	(1.34, 24.03)	0.02
	G	77	39.3	138	32.1	Ref.		
	C	119	60.7	292	67.9	1.75	(1.07, 2.87)	0.03
**rs9994218**	TT	93	94.9	203	94.0	Ref.		
**Intron**	TC	5	5.1	13	6.0	3.16	(0.91, 10.97)	0.07
	CC	0	0.0	0	0.0	-		
	T	191	97.5	419	97.0	Ref.		
	C	5	2.6	13	3.0	3.00	(0.89, 10.05)	0.08
**rs11097262**	TT	13	13.3	18	8.4	Ref.		
**Intron**	CT	51	52.0	90	41.9	3.05	(0.74, 12.53)	0.12
	CC	34	34.7	107	49.8	6.67	(1.56, 28.52)	0.01
	T	77	39.3	126	29.3	Ref.		
	C	119	60.7	304	70.7	2.07	(1.25, 3.44)	0.0046
**rs6852859**	GG	39	39.8	78	36.1	Ref.		
**Intron**	GA	46	46.9	105	48.6	1.27	(0.63, 2.58)	0.50
	AA	13	13.3	33	15.3	1.36	(0.49, 3.79)	0.56
	G	124	63.3	261	60.4	Ref.		
	A	72	36.7	171	39.6	1.19	(0.74, 1.91)	0.48
**rs999361**	TT	17	17.4	30	14.0	Ref.		
**Intron**	GT	55	56.1	106	49.3	1.65	(0.60, 4.58)	0.33
	GG	26	26.5	79	36.7	2.70	(0.90, 8.10)	0.08
	T	89	45.4	166	38.6	Ref.		
	G	107	54.6	264	61.4	1.52	(0.95, 2.43)	0.08
**ANXA2**								
**rs11854079**	AA	31	31.6	95	44.4	Ref.		
**Intron**	AG	53	54.1	86	40.2	0.45	(0.22, 0.94)	0.03
	GG	14	14.3	33	15.4	1.20	(0.45, 3.25)	0.71
	A	115	58.7	276	64.5	Ref.		
	G	81	41.3	152	35.5	0.89	(0.55, 1.44)	0.64
**rs11071521**	GG	32	32.7	95	44.2	Ref.		
**Intron**	GT	52	53.1	84	39.1	0.50	(0.24, 1.03)	0.06
	TT	14	14.3	36	16.7	1.29	(0.48, 3.48)	0.62
	G	116	59.2	274	63.7	Ref.		
	T	80	40.8	156	36.3	0.94	(0.58, 1.52)	0.80
**rs8030787**	CC	34	34.7	74	34.6	Ref.		
**Intron**	CT	55	56.1	99	46.3	0.89	(0.43, 1.86)	0.76
	TT	9	9.2	41	19.2	2.53	(0.90, 7.12)	0.08
	C	123	62.8	247	57.7	Ref.		
	T	73	37.2	181	42.3	1.38	(0.86, 2.22)	0.18

EV71, enterovirus 71; SCARB2, scavenger receptor class B member 2; PSGL-1, P-selectin glycoprotein ligand-1; ANXA2, annexin II.

^a^The ORs, 95% CIs, and P values were calculated by multivariate logistic regression with adjustment for age and gender.

To verify the results from the first cohort, we further tested the association of these SNPs in the second cohort collected from 2011 to 2012 and found that the significant SNPs identified were the same as those found in the first cohort. For the combined cohort of EV71 cases collected from 2000 to 2012, Table 3 shows that the genetic polymorphisms of the EV71 receptor SCARB2 (rs6824953 and rs11097262) may be associated with the susceptibility to EV71 infection by multivariate logistic regression analysis after adjustment for age and gender. For the SCARB2 rs6824953 polymorphism, cases bearing the G allele had an increased susceptibility to EV71 infection compared to those with the C allele (OR 1.62; 95% CI 1.07–2.39, P = 0.02). Similarly, for the SCARB2 rs11097262 polymorphism, cases bearing the C allele had increased susceptibility to EV71 infection compared to those with the T allele (OR 1.64; 95% CI, 1.09–2.47, P = 0.02). There was significant high linkage disequilibrium (LD) between rs6824953 and rs11097262 (r^2^ = 0.88).

**Table 3:** Susceptible host gene polymorphisms in normal cases and the combined cohort of EV71 cases.

Gene	Genotype/	Normal cases (N = 98)		EV71 cases (N = 599)		Logistic regression analysis		
(SNP)	Allele	No.	%	No.	%	OR^a^	(95% CI)^a^	P value^a^
**SCARB2**								
**rs6824953**	CC	15	15.3	64	10.7	Ref.		
**Intron**	GC	53	54.1	255	42.7	1.32	(0.55, 3.16)	0.54
	GG	30	30.6	278	46.6	2.49	(1.00, 6.21)	0.05
	C	83	42.4	383	32.1	Ref.		
	G	113	57.7	811	67.9	1.60	(1.07, 2.39)	0.02
**rs11097262**	TT	13	13.3	57	9.6	Ref.		
**Intron**	CT	51	52.0	256	42.9	1.79	(0.68, 4.72)	0.24
	CC	34	34.7	284	47.6	3.04	(1.13, 8.23)	0.03
	T	77	39.3	370	31.0	Ref.		
	C	119	60.7	824	69.0	1.64	(1.09, 2.47)	0.02

EV71, enterovirus 71; SCARB2, scavenger receptor class B member 2.

^a^The ORs, 95% CIs, and P values were calculated by multivariate logistic regression with adjustment for age and gender.

### Association of polymorphisms with severity of EV71 infection

Table 4 shows the SNPs that were significantly different between the mild group and the severe group in the first cohort collected before 2011. Table 4 lists only SNPs with p values less than 0.10, and all the detailed results are shown in S3 Table. The SNPs in SCARB2 and ANXA2 were not associated with severe EV71 infection, whereas polymorphisms in PSGL-1 (rs7137098 and rs8179137) may be associated with severe CNS involvement. To verify the results from the first cohort, we further tested the association of these SNPs in the second cohort collected from 2011 to 2012, and the significant SNPs were the same as those found in the first cohort.

**Table 4:** Polymorphisms associated with severity of EV71 infection among the first cohort of EV71 cases before 2011.

Gene	Genotype/	Mild group^a^ (N = 152)		Severe group^b^ (N = 65)		Logistic regression analysis		
(SNP)	Allele	No.	%	No.	%	OR^c^	(95% CI)^c^	P value^c^
**SCARB2**								
rs7679797	GG	27	17.9	6	9.2	Ref.	(0.71, 5.23)	0.20
Intron	CG	75	49.7	31	47.7	1.93	(0.91, 6.98)	0.07
	CC	49	32.5	28	43.1	2.52		
	G	129	42.7	43	33.1	Ref.		
	C	173	57.3	87	66.9	1.48	(0.96, 2.31)	0.08
rs13119254	AA	90	59.6	37	56.9	Ref.		
Intron	AG	60	39.7	24	36.9	0.91	(0.49, 1.70)	0.76
	GG	1	0.7	4	6.2	8.14	(0.86, 77.15)	0.07
	A	240	79.5	98	75.4	Ref.		
	G	62	20.5	32	24.6	1.20	(0.73, 1.97)	0.48
rs17001594	AA	35	23.3	8	12.3	Ref.		
Intron	GA	78	52.0	34	52.3	1.86	(0.77, 4.49)	0.17
	GG	37	24.7	23	35.4	2.60	(1.01, 6.69)	0.05
	A	148	49.3	50	38.5	Ref.		
	G	152	50.7	80	61.5	1.53	(0.99, 2.35)	0.05
**ANXA2**								
rs11071521	GG	62	41.3	33	50.8	2.10	(0.83, 5.28)	0.12
Intron	GT	60	40.0	24	36.9	1.55	(0.60, 4.01)	0.37
	TT	28	18.7	8	12.3	Ref.		
	G	184	61.3	90	69.2	1.50	(0.95, 2.35)	0.08
	T	116	38.7	40	30.8	Ref.		
**PSGL-1 (SELPLG)**								
rs2228315	AA	22	14.7	4	6.2	Ref.		
Exon	GA	63	42.0	29	44.6	2.48	(0.76, 8.06)	0.13
	GG	65	43.3	32	49.2	2.67	(0.83, 8.58)	0.10
	A	107	35.7	37	28.5	Ref.		
	G	193	64.3	93	71.5	1.39	(0.88, 2.21)	0.16
rs7137098	TT	63	41.7	20	30.8	Ref.		
Intron	TA	74	49.0	30	46.2	1.24	(0.63, 2.44)	0.53
	AA	14	9.3	15	23.1	2.75	(1.11, 6.81)	0.03
	T	200	66.2	70	53.9	Ref.		
	A	102	33.8	60	46.2	1.55	(1.01, 2.39)	0.04
rs8179137	AA	96	63.6	32	49.2	Ref.		
Intron	AG	52	34.4	27	41.5	1.58	(0.84, 2.99)	0.16
	GG	3	2.0	6	9.2	5.27	(1.17, 23.69)	0.03
	A	244	80.8	91	70.0	Ref.		
	G	58	19.2	39	30.0	1.77	(1.09, 2.88)	0.02

EV71, enterovirus 71; SCARB2, scavenger receptor class B member 2; PSGL-1, P-selectin glycoprotein ligand-1; ANXA2, annexin II.

^a^The mild group included uncomplicated cases and cases with myoclonic jerk or aseptic meningitis.

^b^The severe group included severe CNS cases (encephalitis, polio-like syndrome or encephalomyelitis) with or without cardiopulmonary failure.

^c^The ORs, 95% CIs, and P values were calculated by multivariate logistic regression with adjustment for age and gender.

Table 5 shows the SNPs that were significantly different among all EV71 cases with different clinical severities. We found that genetic polymorphisms in the EV71 receptor PSGL-1 (rs7137098 and rs8179137) may be associated with the severity of EV71 infection. Multivariate logistic regression analyses performed by adjusting for age and sex revealed that PSGL-1 polymorphisms (rs7137098 and rs8179137) were significantly associated with severe EV71 CNS involvement. For the PSGL-1 rs7137098 polymorphism, cases bearing the A allele had an increased risk of severe EV71 CNS infection compared with those bearing the G allele (OR 1.46; 95% CI 1.1–1.96). Similarly, for the PSGL-1 rs8179137 polymorphism, cases with the A allele also had significantly higher risk of severe EV71 CNS infection than those carrying the G allele (OR 1.47; 95% CI 1.07–2.03). In addition, no significant linkage disequilibrium was found between rs7137098 and rs8179137 (r^2^ = 0).

**Table 5:** Polymorphisms associated with severity of EV71 infection among the combined cohort of EV71 cases.

Gene	Genotype/	Mild group^a^ (N = 482)		Severe group^b^ (N = 117)		Logistic regression analysis		
(SNP)	Allele	No.	%	No.	%	OR^c^	(95% CI)^c^	P value^c^
**PSGL-1 (SELPLG)**								
rs7137098	TT	202	42.0	36	30.8	Ref.		
Intron	TA	227	47.2	60	51.3	1.48	(0.94, 2.34)	0.09
	AA	52	10.8	21	18.0	2.23	(1.20, 4.15)	0.01
	T	631	65.6	132	56.4	Ref.		
	A	331	34.4	102	43.6	1.46	(1.10, 1.96)	0.01
rs8179137	AA	285	59.3	57	48.7	Ref.		
Intron	AG	175	36.4	50	42.7	1.45	(0.95, 2.22)	0.09
	GG	21	4.4	10	8.6	2.36	(1.05, 5.29)	0.04
	A	745	77.4	164	70.1	Ref.		
	G	217	22.6	70	29.9	1.47	(1.07, 2.03)	0.02

EV71, enterovirus 71; PSGL-1, P-selectin glycoprotein ligand-1.

^a^The mild group including uncomplicated cases and cases with myoclonic jerk or aseptic meningitis.

^b^The severe group including severe CNS cases (encephalitis, polio-like syndrome or encephalomyelitis) with or without cardiopulmonary failure.

^c^The ORs, 95% CIs, and p values were calculated by multivariate logistic regression with adjustment for age and gender.

## Discussion

Our study found that genetic polymorphisms in SCARB2 (rs6824953 and rs11097262) may play a role in susceptibility to EV71 infection in the Taiwanese population. Additionally, genetic polymorphisms in PSGL-1 (rs7137098 and rs8179137) were associated with severe EV71 infection. To the best of our knowledge, this study is the first to demonstrate associations of these SNPs with EV71 infection susceptibility and severe CNS involvement, confirming the initial hypothesis that SCARB2 and PSGL-1 may play a role in the pathogenesis of this important infection.

The genetic association between SCARB2 polymorphisms and EV71 infection is biologically plausible. Yamayoshi et al. identified SCARB2 as an EV71 receptor on RD cells [22], which have been widely used for the isolation of EV71 from clinical specimens. This protein is a type III glycoprotein that is located primarily in the membranes of lysosomes and endosomes. Several studies in humans have shown that this protein is ubiquitously expressed and is involved in the pathogenesis of HFMD (hand-foot-and-mouth disease) caused by EV71 and possibly by coxsackievirus A16 [22,32,33]. Human SCARB2 has 10 potential N-glycosylation sites [24], but the carbohydrate chains of human SCARB2 are not essential for the interaction between it and EV71 [34]. Experiments using a series of chimeric proteins between human and mouse SCARB2 identified that amino acids 142–204 of human SCARB2 (encoded by human SCARB2 exon 4) are responsible for EV71 binding and infection [34]. However, we did not find significant differences in SNP frequency within the coding region of amino acids 142–204 when comparing EV71 cases and controls. Instead, the genetic polymorphisms in SCARB2 (rs6824953 and rs11097262) that were associated with susceptibility were both located in intronic areas of the SCARB2 gene. We hypothesize that these SNPs may regulate the function or expression of SCARB2 and thus affect EV71 susceptibility, but further studies are needed to clarify the mechanism.

Yamayoshi et al. reported that EV71 could infect mouse L cells expressing SCARB2 more efficiently than those expressing PSGL-1. The difference in the binding capacities of the two receptors was not the sole determinant of infection efficiency. In addition to viral binding and internalization, SCARB2 is capable of viral uncoating, whereas PSGL-1 is not, which explains why mouse L cells expressing PSGL-1 have a lower infection EV71 efficiency of than those expressing SCARB2 [35]. Our results show that polymorphisms in SCARB2, rather than those in PSGL-1, were associated with EV71 susceptibility, which also implicates SCARB2 in a more important role in infection efficiency and illustrates how genetic variation in SCARB2 can affect host susceptibility.

By contrast, polymorphisms in PSGL-1 (rs7137098 and rs8179137), rather than those in SCARB2, were associated with severe EV71 CNS involvement. During EV71 outbreaks in Taiwan, individuals with severe EV71-associated encephalitis and cardiopulmonary failure showed a marked depletion of T cells but had high levels of proinflammatory cytokines [15,36]. Because of this T cell involvement, Nishimura et al. generated a retroviral complementary DNA (cDNA) library from Jurkat T cells that are susceptible to EV71 infection and used it for expression cloning to identify PSGL-1 as the receptor that specifically binds EV71 virions [23]. PSGL-1 is expressed on white blood cells and functions as a high affinity counterreceptor for the cell adhesion molecules P-, E- and L- selectin, which are expressed on myeloid cells and stimulated T lymphocytes. As such, this protein plays a critical role in leukocyte trafficking during inflammation by tethering leukocytes to activated platelets or to endothelia expressing selectins. Our study found that genetic polymorphisms in intronic regions of PSGL-1 (rs7137098 and rs8179137) are associated with severe EV71 CNS involvement, similar to cases in which significant leukocytosis and proinflammatory cytokine storm were observed in previous studies [15,36]. The above phenomena may be correlated, and PSGL-1 polymorphisms may be involved in CNS pathogenesis.

It was well known that ANXA2, a member of the annexin family that is expressed on the surface of endothelial cells, is a specific factor involved in the early stages of EV71 infection [29]. Binding of EV71 to ANXA2 on the cell surface can enhance viral entry and infectivity, especially at a low infective dose [29]. In addition, Lui et al. found that EV71 infection may be actin dependent and involve the actin-binding protein ANXA2 [37]. However, we did not find an association of ANXA2 polymorphisms with susceptibility, severity, or clinical outcomes of EV71 infections in our study. This might indicate that ANXA2 polymorphisms play a less important role in infection efficiency and host susceptibility than SCARB2 and PSGL-1 polymorphisms. In addition, the sample size of our study was relatively small and may not have been large enough to determine the significance of ANXA2 polymorphisms. The detailed mechanism underling this observation warrants further studies.

There were some limitations in this study. First, the sample size was not large; therefore, it was difficult to find significant differences among low frequency SNPs. Second, we did not evaluate SCARB2 rs6825004 in the combined cohort because the linkage disequilibrium (LD) value between rs6824953 and rs6825004 was very high (D' = 1, r-square = 1), and they were highly correlated. Therefore, we only tested rs6824953, not rs6825004. Third, the functions of the significant SNPs in this study have not been clarified, and the detailed mechanism needs to be further explored. Therefore, the SCARB2 and PSGL-1 sequence variants, the signaling pathway involved in the novel regulatory effects of these polymorphisms in the SCARB2 and PSGL-1 genes, and their functional influence on the clinical consequences of EV71 infection should be further investigated.

## Conclusions

Our study revealed that genetic polymorphisms in SCARB2 (rs6824953 and rs11097262) could play a role in susceptibility to EV71 infection in a Taiwan population, and genetic polymorphisms in PSGL-1 (rs7137098 and rs8179137) are associated with clinical severity of EV71 infection.

## Supporting information

S1 Table List of SNPs in SCARB2, SELPLG, ANXA2 tested in this study. (DOCX)

S2 Table Susceptible host gene polymorphisms in normal cases and EV71 cases before 2011. (DOCX)

S3 Table Polymorphisms associated with severity of EV71 infection among the first cohort of EV71 cases before 2011. (DOCX)
