# Supplementary material for: Polymorphisms in enterovirus 71 receptors associated with susceptibility and clinical severity
Source: PLoS One. 2018 Nov 5;13(11):e0206769. doi: 10.1371/journal.pone.0206769 (PMC6218064; doi:10.1371/journal.pone.0206769)
Supplement: S1 Table — (DOCX) [file pone.0206769.s001.docx]

**S1 Table. List of SNPs of SCARB2, SELPLG, ANXA2 tested in this study.**

| SNP rs# | Gene(s) | Alleles | SNP position | Function | AA change |
| --- | --- | --- | --- | --- | --- |
| rs77814624  rs7679797  rs17001559  rs13119254  rs3796498  rs112779426  rs7676834  rs73826386  rs17001594  rs6824953  rs6825004  rs17001606  rs9994218  rs11097262  rs6852859  rs1542093  rs999361  rs17001622  rs17001640  rs12508946  rs17001659  rs12640238  rs35624925  rs146560583  rs143655258  rs147159813  rs117600063  rs2228315  rs7137098  rs8179137  rs3782522  rs3782520  rs7163836  rs11854079  rs11629852  rs11071521  rs8030787  rs1551347  rs4775262  rs9920823  rs17237276  rs16942555  rs8033800 | SCARB2  SCARB2  SCARB2  SCARB2  SCARB2  SCARB2  SCARB2  SCARB2  SCARB2  SCARB2  SCARB2  SCARB2  SCARB2  SCARB2  SCARB2  SCARB2  SCARB2  SCARB2  SCARB2  SCARB2  SCARB2  SCARB2  SCARB2  SCARB2  SCARB2  SCARB2  SCARB2  SELPLG  SELPLG  SELPLG  SELPLG  SELPLG  ANXA2  ANXA2  ANXA2  ANXA2  ANXA2  ANXA2  ANXA2  ANXA2  ANXA2  ANXA2  ANXA2 | C/T  C/G  A/G  A/G  A/G  A/G  C/G  C/T  A/G  C/G  C/G  A/T  C/T  C/T  A/G  A/G  G/T  A/C  A/G  A/T  C/T  G/T  -/C  A/G  C/T  C/T  A/T  A/G  A/T  A/G  A/G  A/G  C/T  A/G  G/T  G/T  C/T  A/G  C/T  G/T  A/G  G/T  A/T | chr4:77089557  chr4:77089928  chr4:77095048  chr4:77095143  chr4:77095940  chr4:77097617  chr4:77101637  chr4:77102168  chr4:77107562  chr4:77110311  chr4:77110365  chr4:77111481  chr4:77112851  chr4:77114936  chr4:77115151  chr4:77116143  chr4:77117574  chr4:77119218  chr4:77124745  chr4:77127771  chr4:77131069  chr4:77132557  chr4:77100676  chr4:77100693  chr4:77100807  chr4:77100837  chr4:77100852  chr12:109017898  chr12:109018418  chr12:109019691  chr12:109024725  chr12:109027340  chr15:60640228  chr15:60643068  chr15:60650015  chr15:60655467  chr15:60664030  chr15:60666228  chr15:60666686  chr15:60673849  chr15:60685974  chr15:60687152  chr15:60689179 | exon  intron  intron  intron  intron  exon  intron  exon  intron  intron  intron  intron  intron  intron  intron  intron  intron  intron  intron  intron  intron  intron  exon  exon  exon  exon  exon  exon  intron  intron  intron  intron  intron  intron  intron  intron  intron  intron  intron  intron  intron  intron  intron | Val396Ile  Thr226Ile  Arg121Gln  Phe202Cys  Pro197Ser  Met159Val  Val149Met  Ile144Leu  Met62Ile |

SNP, single nucleotide polymorphism; SCARB2, scavenger receptor class B member 2; PSGL-1, P-selectin glycoprotein ligand-1; ANXA2, annexin II; AA, amino acid.
