# Supplementary material for: Polymorphisms in enterovirus 71 receptors associated with susceptibility and clinical severity
Source: PLoS One. 2018 Nov 5;13(11):e0206769. doi: 10.1371/journal.pone.0206769 (PMC6218064; doi:10.1371/journal.pone.0206769)
Supplement: S2 Table — (DOCX) [file pone.0206769.s002.docx]

**S2 Table. Susceptible host gene polymorphisms in normal cases and EV71 cases before 2011**

| Gene | Genotype/ | Normal cases (N=98) | | EV71 cases (N=217) | | Logistic regression analysis | | |
| --- | --- | --- | --- | --- | --- | --- | --- | --- |
| (SNP) | Allele | No. | % | No. | % | OR^a^ | (95% CI)^a^ | P value^a^ |
| **SCARB2** | |  |  |  |  |  |  |  |
| rs77814624 | CC | 98 | 100.0 | 217 | 100.0 |  |  |  |
| exon | CT | 0 | 0.0 | 0 | 0.0 |  |  |  |
|  | TT | 0 | 0.0 | 0 | 0.0 |  |  |  |
|  | C | 196 | 100.0 | 432 | 100.0 |  |  |  |
|  | T | 0 | 0.0 | 0 | 0.0 |  |  |  |
| rs7679797 | CC | 40 | 40.8 | 77 | 35.7 | Ref. |  |  |
| intron | CG | 45 | 45.9 | 106 | 49.1 | 1.44 | (0.71, 2.93) | 0.31 |
|  | GG | 13 | 13.3 | 33 | 15.3 | 1.46 | (0.52, 4.08) | 0.47 |
|  | C | 125 | 63.8 | 260 | 60.2 | Ref. |  |  |
|  | G | 71 | 36.2 | 172 | 39.8 | 1.25 | (0.78, 2.02) | 0.35 |
| rs17001559 | AA | 90 | 91.8 | 188 | 87.0 | Ref. |  |  |
| intron | AG | 8 | 8.2 | 28 | 13.0 | 1.27 | (0.42, 3.84) | 0.67 |
|  | GG | 0 | 0.0 | 0 | 0.0 | - |  |  |
|  | A | 188 | 95.9 | 404 | 93.5 | Ref. |  |  |
|  | G | 8 | 4.1 | 28 | 6.5 | 1.26 | (0.43, 3.68) | 0.68 |
| rs13119254 | AA | 66 | 67.4 | 127 | 58.8 | Ref. |  |  |
| intron | AG | 30 | 30.6 | 84 | 38.9 | 1.62 | (0.81, 3.25) | 0.17 |
|  | GG | 2 | 2.0 | 5 | 2.3 | 2.46 | (0.32, 19.18) | 0.39 |
|  | A | 162 | 82.7 | 338 | 78.2 | Ref. |  |  |
|  | G | 34 | 17.4 | 94 | 21.8 | 1.52 | (0.86, 2.69) | 0.15 |
| rs3796498 | GG | 77 | 78.6 | 158 | 73.5 | 1.27 | (0.08, 21.49) | 0.87 |
| intron | GA | 20 | 20.4 | 54 | 25.1 | 1.12 | (0.06, 20.56) | 0.94 |
|  | AA | 1 | 1.0 | 3 | 1.4 | Ref. |  |  |
|  | G | 174 | 88.8 | 370 | 86.1 | 1.13 | (0.54, 2.39) | 0.74 |
|  | A | 22 | 11.2 | 60 | 14.0 | Ref. |  |  |
| rs112779426 | GG | 98 | 100.0 | 216 | 100.0 |  |  |  |
| exon | GA | 0 | 0.0 | 0 | 0.0 |  |  |  |
|  | AA | 0 | 0.0 | 0 | 0.0 |  |  |  |
|  | G | 196 | 100.0 | 432 | 100.0 |  |  |  |
|  | A | 0 | 0.0 | 0 | 0.0 |  |  |  |
| rs146560583 | GG | 98 | 100.0 | 216 | 100.0 |  |  |  |
| exon | GA | 0 | 0.0 | 0 | 0.0 |  |  |  |
|  | AA | 0 | 0.0 | 0 | 0.0 |  |  |  |
|  | G | 196 | 100.0 | 432 | 100.0 |  |  |  |
|  | A | 0 | 0.0 | 0 | 0.0 |  |  |  |
| rs143655258 | TT | 98 | 100.0 | 216 | 100.0 |  |  |  |
| exon | TC | 0 | 0.0 | 0 | 0.0 |  |  |  |
|  | CC | 0 | 0.0 | 0 | 0.0 |  |  |  |
|  | T | 196 | 100.0 | 432 | 100.0 |  |  |  |
|  | C | 0 | 0.0 | 0 | 0.0 |  |  |  |
| rs147159813 | CC | 98 | 100.0 | 216 | 100.0 |  |  |  |
| exon | CT | 0 | 0.0 | 0 | 0.0 |  |  |  |
|  | TT | 0 | 0.0 | 0 | 0.0 |  |  |  |
|  | C | 196 | 100.0 | 432 | 100.0 |  |  |  |
|  | T | 0 | 0.0 | 0 | 0.0 |  |  |  |
| rs117600063 | TT | 97 | 99.0 | 213 | 98.6 | Ref. |  |  |
| exon | TA | 1 | 1.0 | 3 | 1.4 | 4.72 | (0.40, 55.45) | 0.22 |
|  | AA | 0 | 0.0 | 0 | 0.0 | - |  |  |
|  | T | 195 | 99.5 | 429 | 99.3 | Ref. |  |  |
|  | A | 1 | 0.5 | 3 | 0.7 | 4.61 | (0.40, 53.14) | 0.22 |
| rs7676834 | CC | 40 | 40.8 | 80 | 37.0 | Ref. |  |  |
| intron | CG | 45 | 45.9 | 103 | 47.7 | 1.31 | (0.65, 2.65) | 0.45 |
|  | GG | 13 | 13.3 | 33 | 15.3 | 1.38 | (0.49, 3.84) | 0.54 |
|  | C | 125 | 63.8 | 263 | 60.9 | Ref. |  |  |
|  | G | 71 | 36.2 | 169 | 39.1 | 1.20 | (0.75, 1.94) | 0.44 |
| rs73826386 | CC | 98 | 100.0 | 216 | 100.0 |  |  |  |
| exon | CT | 0 | 0.0 | 0 | 0.0 |  |  |  |
|  | TT | 0 | 0.0 | 0 | 0.0 |  |  |  |
|  | C | 196 | 100.0 | 432 | 100.0 |  |  |  |
|  | T | 0 | 0.0 | 0 | 0.0 |  |  |  |
| rs17001594 | GG | 37 | 37.8 | 60 | 27.9 | Ref. |  |  |
| intron | GA | 43 | 43.9 | 112 | 52.1 | 2.06 | (0.98, 4.35) | 0.06 |
|  | AA | 18 | 18.4 | 43 | 20.0 | 1.31 | (0.49, 3.51) | 0.60 |
|  | G | 117 | 59.7 | 232 | 54.0 | Ref. |  |  |
|  | A | 79 | 40.3 | 198 | 46.1 | 1.25 | (0.78, 1.99) | 0.35 |
| rs6824953 | CC | 15 | 15.3 | 22 | 10.2 | Ref. |  |  |
| intron | GC | 53 | 54.1 | 86 | 40.0 | 1.23 | (0.39, 3.83) | 0.72 |
|  | GG | 30 | 30.6 | 107 | 49.8 | 3.31 | (1.03, 10.64) | 0.04 |
|  | C Allele | 83 | 42.4 | 130 | 30.2 | Ref. |  |  |
|  | G Allele | 113 | 57.7 | 300 | 69.8 | 1.94 | (1.19, 3.17) | 0.01 |
| rs6825004 | GG | 13 | 13.3 | 19 | 8.8 | Ref. |  |  |
| intron | CG | 51 | 52.0 | 100 | 46.5 | 3.42 | (0.85, 13.75) | 0.08 |
|  | CC | 34 | 34.7 | 96 | 44.7 | 5.68 | (1.34, 24.03) | 0.02 |
|  | G | 77 | 39.3 | 138 | 32.1 | Ref. |  |  |
|  | C | 119 | 60.7 | 292 | 67.9 | 1.75 | (1.07, 2.87) | 0.03 |
| rs17001606 | AA | 90 | 91.8 | 187 | 87.0 | Ref. |  |  |
| intron | AT | 8 | 8.2 | 28 | 13.0 | 1.27 | (0.42, 3.84) | 0.67 |
|  | TT | 0 | 0.0 | 0 | 0.0 | - |  |  |
|  | A | 188 | 95.9 | 402 | 93.5 | Ref. |  |  |
|  | T | 8 | 4.1 | 28 | 6.5 | 1.26 | (0.43, 3.68) | 0.68 |
| rs9994218 | TT | 93 | 94.9 | 203 | 94.0 | Ref. |  |  |
| intron | TC | 5 | 5.1 | 13 | 6.0 | 3.16 | (0.91, 10.97) | 0.07 |
|  | CC | 0 | 0.0 | 0 | 0.0 | - |  |  |
|  | T | 191 | 97.5 | 419 | 97.0 | Ref. |  |  |
|  | C | 5 | 2.6 | 13 | 3.0 | 3.00 | (0.89, 10.05) | 0.08 |
| rs11097262 | TT | 13 | 13.3 | 18 | 8.4 | Ref. |  |  |
| intron | CT | 51 | 52.0 | 90 | 41.9 | 3.05 | (0.74, 12.53) | 0.12 |
|  | CC | 34 | 34.7 | 107 | 49.8 | 6.67 | (1.56, 28.52) | 0.01 |
|  | T | 77 | 39.3 | 126 | 29.3 | Ref. |  |  |
|  | C | 119 | 60.7 | 304 | 70.7 | 2.07 | (1.25, 3.44) | 0.0046 |
| rs6852859 | GG | 39 | 39.8 | 78 | 36.1 | Ref. |  |  |
| intron | GA | 46 | 46.9 | 105 | 48.6 | 1.27 | (0.63, 2.58) | 0.50 |
|  | AA | 13 | 13.3 | 33 | 15.3 | 1.36 | (0.49, 3.79) | 0.56 |
|  | G | 124 | 63.3 | 261 | 60.4 | Ref. |  |  |
|  | A | 72 | 36.7 | 171 | 39.6 | 1.19 | (0.74, 1.91) | 0.48 |
| rs1542093 | AA | 90 | 91.8 | 188 | 87.0 | Ref. |  |  |
| intron | AG | 8 | 8.2 | 28 | 13.0 | 1.27 | (0.42, 3.84) | 0.67 |
|  | GG | 0 | 0.0 | 0 | 0.0 | - |  |  |
|  | A | 188 | 95.9 | 404 | 93.5 | Ref. |  |  |
|  | G | 8 | 4.1 | 28 | 6.5 | 1.26 | (0.43, 3.68) | 0.68 |
| rs999361 | TT | 17 | 17.4 | 30 | 14.0 | Ref. |  |  |
| intron | GT | 55 | 56.1 | 106 | 49.3 | 1.65 | (0.60, 4.58) | 0.33 |
|  | GG | 26 | 26.5 | 79 | 36.7 | 2.70 | (0.90, 8.10) | 0.08 |
|  | T | 89 | 45.4 | 166 | 38.6 | Ref. |  |  |
|  | G | 107 | 54.6 | 264 | 61.4 | 1.52 | (0.95, 2.43) | 0.08 |
| rs17001622 | CC | 41 | 41.8 | 79 | 36.7 | Ref. |  |  |
| intron | CA | 44 | 44.9 | 104 | 48.4 | 1.44 | (0.71, 2.91) | 0.31 |
|  | AA | 13 | 13.3 | 32 | 14.9 | 1.28 | (0.45, 3.64) | 0.64 |
|  | C | 126 | 64.3 | 262 | 60.9 | Ref. |  |  |
|  | A | 70 | 35.7 | 168 | 39.1 | 1.20 | (0.75, 1.94) | 0.45 |
| rs17001640 | AA | 34 | 34.7 | 66 | 30.6 | Ref. |  |  |
| intron | AG | 49 | 50.0 | 104 | 48.2 | 0.95 | (0.45, 1.98) | 0.89 |
|  | GG | 15 | 15.3 | 46 | 21.3 | 2.10 | (0.81, 5.45) | 0.13 |
|  | A | 117 | 59.7 | 236 | 54.6 | Ref. |  |  |
|  | G | 79 | 40.3 | 296 | 45.4 | 1.36 | (0.86, 2.17) | 0.19 |
| rs12508946 | TT | 90 | 91.8 | 180 | 83.7 | Ref. |  |  |
| intron | TA | 8 | 8.2 | 32 | 14.9 | 2.07 | (0.74, 5.79) | 0.17 |
|  | AA | 0 | 0.0 | 3 | 1.4 | - |  |  |
|  | T | 188 | 95.9 | 392 | 91.2 | Ref. |  |  |
|  | A | 8 | 4.1 | 38 | 8.8 | 2.02 | (0.76, 5.43) | 0.16 |
| rs17001659 | CC | 12 | 12.2 | 17 | 7.9 | Ref. |  |  |
| intron | TC | 37 | 37.8 | 103 | 47.9 | 1.71 | (0.59, 4.99) | 0.32 |
|  | TT | 49 | 50.0 | 95 | 44.2 | 1.37 | (0.47, 3.99) | 0.57 |
|  | C | 61 | 31.1 | 137 | 31.9 | Ref. |  |  |
|  | T | 135 | 68.9 | 293 | 68.1 | 1.04 | (0.64, 1.70) | 0.87 |
| rs12640238 | GG | 12 | 12.2 | 18 | 8.3 | Ref. |  |  |
| intron | TG | 37 | 37.8 | 103 | 47.7 | 1.72 | (0.59, 5.03) | 0.32 |
|  | TT | 49 | 50.0 | 95 | 44.0 | 1.37 | (0.47, 4.01) | 0.56 |
|  | G | 61 | 31.1 | 139 | 32.2 | Ref. |  |  |
|  | T | 135 | 68.9 | 293 | 67.8 | 1.04 | (0.64, 1.70) | 0.87 |
| **ANXA2** |  |  |  |  |  |  |  |  |
| rs7163836 | CC | 22 | 22.5 | 42 | 19.6 | Ref. |  |  |
| intron | CT | 59 | 60.2 | 106 | 49.5 | 0.65 | (0.28, 1.50) | 0.31 |
|  | TT | 17 | 17.4 | 66 | 30.8 | 1.52 | (0.59, 3.97) | 0.39 |
|  | C | 103 | 52.6 | 190 | 44.4 | Ref. |  |  |
|  | T | 93 | 47.4 | 238 | 55.6 | 1.25 | (0.78, 1.98) | 0.35 |
| rs11854079 | AA | 31 | 31.6 | 95 | 44.4 | Ref. |  |  |
| intron | AG | 53 | 54.1 | 86 | 40.2 | 0.45 | (0.22, 0.94) | 0.03 |
|  | GG | 14 | 14.3 | 33 | 15.4 | 1.20 | (0.45, 3.25) | 0.71 |
|  | A | 115 | 58.7 | 276 | 64.5 | Ref. |  |  |
|  | G | 81 | 41.3 | 152 | 35.5 | 0.89 | (0.55, 1.44) | 0.64 |
| rs11629852 | TT | 64 | 65.3 | 145 | 67.8 | 2.13 | (0.48, 9.40) | 0.32 |
| intron | TG | 29 | 29.6 | 55 | 25.7 | 1.77 | (0.38, 8.27) | 0.47 |
|  | GG | 5 | 5.1 | 14 | 6.5 | Ref. |  |  |
|  | T | 157 | 80.1 | 345 | 80.6 | 1.35 | (0.76, 2.43) | 0.31 |
|  | G | 39 | 19.9 | 83 | 19.4 | Ref. |  |  |
| rs11071521 | GG | 32 | 32.7 | 95 | 44.2 | Ref. |  |  |
| intron | GT | 52 | 53.1 | 84 | 39.1 | 0.50 | (0.24, 1.03) | 0.06 |
|  | TT | 14 | 14.3 | 36 | 16.7 | 1.29 | (0.48, 3.48) | 0.62 |
|  | G | 116 | 59.2 | 274 | 63.7 | Ref. |  |  |
|  | T | 80 | 40.8 | 156 | 36.3 | 0.94 | (0.58, 1.52) | 0.80 |
| rs8030787 | CC | 34 | 34.7 | 74 | 34.6 | Ref. |  |  |
| intron | CT | 55 | 56.1 | 99 | 46.3 | 0.89 | (0.43, 1.86) | 0.76 |
|  | TT | 9 | 9.2 | 41 | 19.2 | 2.53 | (0.90, 7.12) | 0.08 |
|  | C | 123 | 62.8 | 247 | 57.7 | Ref. |  |  |
|  | T | 73 | 37.2 | 181 | 42.3 | 1.38 | (0.86, 2.22) | 0.18 |
| rs1551347 | AA | 54 | 55.1 | 104 | 48.6 | Ref. |  |  |
| intron | AG | 35 | 35.7 | 96 | 44.9 | 1.20 | (0.60, 2.83) | 0.60 |
|  | GG | 9 | 9.2 | 14 | 6.5 | 0.93 | (0.26, 3.40) | 0.91 |
|  | A | 143 | 73.0 | 304 | 71.0 | Ref. |  |  |
|  | G | 53 | 27.0 | 124 | 29.0 | 1.07 | (0.63, 1.79) | 0.81 |
| rs4775262 | CC | 67 | 68.4 | 139 | 65.0 | Ref. |  |  |
| intron | CT | 27 | 27.6 | 68 | 31.8 | 1.21 | (0.58, 2.55) | 0.61 |
|  | TT | 4 | 4.1 | 7 | 3.3 | 2.58 | (0.52, 12.74) | 0.24 |
|  | C | 161 | 82.1 | 346 | 80.8 | Ref. |  |  |
|  | T | 35 | 17.9 | 82 | 19.2 | 1.40 | (0.76, 2.56) | 0.28 |
| rs9920823 | GG | 58 | 59.2 | 137 | 63.7 | Ref. |  |  |
| intron | GT | 35 | 35.7 | 68 | 31.6 | 1.11 | (0.55, 2.23) | 0.77 |
|  | TT | 5 | 5.1 | 10 | 4.7 | 1.26 | (0.30, 5.33) | 0.75 |
|  | G | 151 | 77.0 | 342 | 79.5 | Ref. |  |  |
|  | T | 45 | 23.0 | 88 | 20.5 | 1.12 | (0.65, 1.92) | 0.69 |
| rs17237276 | GG | 90 | 91.8 | 193 | 89.8 | Ref. |  |  |
| intron | GA | 8 | 8.2 | 21 | 9.8 | 2.25 | (0.73, 6.93) | 0.16 |
|  | AA | 0 | 0.0 | 1 | 0.5 | - |  |  |
|  | G | 188 | 95.9 | 407 | 94.7 | Ref. |  |  |
|  | A | 8 | 4.1 | 23 | 5.4 | 2.13 | (0.72, 6.29) | 0.17 |
| rs16942555 | TT | 79 | 80.6 | 180 | 84.1 | Ref. |  |  |
| intron | TG | 18 | 18.4 | 33 | 15.4 | 1.23 | (0.53, 2.85) | 0.64 |
|  | GG | 1 | 1.0 | 1 | 0.5 | - |  |  |
|  | T | 176 | 89.8 | 393 | 91.8 | Ref. |  |  |
|  | G | 20 | 10.2 | 35 | 8.2 | 1.12 | (0.51, 2.44) | 0.78 |
| rs8033800 | AA | 66 | 67.4 | 145 | 67.1 | Ref. |  |  |
| intron | AT | 31 | 31.6 | 64 | 29.6 | 1.20 | (0.60, 2.41) | 0.60 |
|  | TT | 1 | 1.0 | 7 | 3.2 | 0.13 | (0.00, 12.55) | 0.39 |
|  | A | 163 | 83.2 | 354 | 81.9 | Ref. |  |  |
|  | T | 33 | 16.8 | 78 | 18.1 | 1.07 | (0.58, 1.96) | 0.83 |
| **PSGL-1 (SELPLG)** | |  |  |  |  |  |  |  |
| rs2228315 | GG | 55 | 56.1 | 97 | 45.1 | Ref. |  |  |
| exon | GA | 34 | 34.7 | 92 | 42.8 | 1.47 | (0.73, 2.94) | 0.28 |
|  | AA | 9 | 9.2 | 26 | 12.1 | 1.39 | (0.46, 4.24) | 0.56 |
|  | G | 144 | 73.5 | 286 | 66.5 | Ref. |  |  |
|  | A | 52 | 26.5 | 144 | 33.4 | 1.29 | (0.78, 2.13) | 0.32 |
| rs7137098 | TT | 39 | 39.8 | 83 | 38.4 | Ref. |  |  |
| intron | TA | 46 | 46.9 | 104 | 48.2 | 1.09 | (0.53, 2.24) | 0.82 |
|  | AA | 13 | 13.3 | 29 | 13.4 | 1.73 | (0.66, 4.59) | 0.27 |
|  | T | 124 | 63.3 | 270 | 62.5 | Ref. |  |  |
|  | A | 72 | 36.7 | 162 | 37.5 | 1.27 | (0.80, 2.03) | 0.32 |
| rs8179137 | AA | 63 | 64.3 | 128 | 59.3 | Ref. |  |  |
| intron | AG | 27 | 27.6 | 79 | 36.6 | 0.95 | (0.44, 2.02) | 0.88 |
|  | GG | 8 | 8.2 | 9 | 4.2 | 1.55 | (0.49, 4.88) | 0.45 |
|  | A | 153 | 78.1 | 335 | 77.6 | Ref. |  |  |
|  | G | 43 | 21.9 | 97 | 22.5 | 1.16 | (0.68, 2.01) | 0.59 |
| rs3782522 | GG | 32 | 32.7 | 65 | 30.2 | Ref. |  |  |
| intron | GA | 47 | 48.0 | 111 | 51.6 | 1.07 | (0.51, 2.26) | 0.86 |
|  | AA | 19 | 19.4 | 39 | 18.1 | 1.27 | (0.51, 3.15) | 0.61 |
|  | G | 111 | 56.6 | 241 | 56.1 | Ref. |  |  |
|  | A | 85 | 43.4 | 189 | 44.0 | 1.13 | (0.71, 1.79) | 0.62 |
| rs3782520 | GG | 88 | 89.8 | 199 | 93.0 | Ref. |  |  |
| intron | GA | 10 | 10.2 | 14 | 6.5 | 1.26 | (0.45, 3.54) | 0.66 |
|  | AA | 0 | 0.0 | 1 | 0.5 | - |  |  |
|  | G | 186 | 94.9 | 412 | 96.3 | Ref. |  |  |
|  | A | 10 | 5.1 | 16 | 3.7 | 1.59 | (0.62, 4.09) | 0.34 |

EV71, enterovirus 71; SCARB2, scavenger receptor class B member 2; PSGL-1, P-selectin glycoprotein ligand-1; ANXA2, annexin II.

^a^The ORs, 95% CI, and P values were calculated by multivariate logistic regression with adjustment of age and gender.
