# Supplementary material for: Polymorphisms in enterovirus 71 receptors associated with susceptibility and clinical severity
Source: PLoS One. 2018 Nov 5;13(11):e0206769. doi: 10.1371/journal.pone.0206769 (PMC6218064; doi:10.1371/journal.pone.0206769)
Supplement: S3 Table — (DOCX) [file pone.0206769.s003.docx]

**S3 Table. Polymorphisms associated with severity of EV71 infection among the first cohort of EV71 cases before 2011.**

|  | Genotype/ | Mild group^a^ (N=152) | |  | Severe group^b^ (N=65) | | Logistic regression analysis | | |
| --- | --- | --- | --- | --- | --- | --- | --- | --- | --- |
|  | Allele | No. | % |  | No. | % | OR^c^ | (95% CI)^c^ | P value^c^ |
| **SCARB2** |  |  |  |  |  |  |  |  |  |
| rs77814624 | CC | 151 | 100.0 |  | 65 | 100.0 |  |  |  |
| exon | CT | 0 | 0.0 |  | 0 | 0.0 |  |  |  |
|  | TT | 0 | 0.0 |  | 0 | 0.0 |  |  |  |
|  | C | 302 | 100.0 |  | 130 | 100.0 |  |  |  |
|  | T | 0 | 0.0 |  | 0 | 0.0 |  |  |  |
| rs7679797 | GG | 27 | 17.9 |  | 6 | 9.2 | Ref. |  |  |
| intron | CG | 75 | 49.7 |  | 31 | 47.7 | 1.93 | (0.71, 5.23) | 0.20 |
|  | CC | 49 | 32.5 |  | 28 | 43.1 | 2.52 | (0.91, 6.98) | 0.07 |
|  | G | 129 | 42.7 |  | 43 | 33.1 | Ref. |  |  |
|  | C | 173 | 57.3 |  | 87 | 66.9 | 1.48 | (0.96, 2.31) | 0.08 |
| rs13119254 | AA | 90 | 59.6 |  | 37 | 56.9 | Ref. |  |  |
| intron | AG | 60 | 39.7 |  | 24 | 36.9 | 0.91 | (0.49, 1.70) | 0.76 |
|  | GG | 1 | 0.7 |  | 4 | 6.2 | 8.14 | (0.86, 77.15) | 0.07 |
|  | A | 240 | 79.5 |  | 98 | 75.4 | Ref. |  |  |
|  | G | 62 | 20.5 |  | 32 | 24.6 | 1.20 | (0.73, 1.97) | 0.48 |
| rs3796498 | AA | 2 | 1.3 |  | 1 | 1.5 | Ref. |  |  |
| intron | GA | 38 | 25.3 |  | 16 | 24.6 | 1.00 | (0.50, 2.01) | 0.99 |
|  | GG | 110 | 73.3 |  | 48 | 73.9 | 1.60 | (0.13, 20.08) | 0.71 |
|  | A | 42 | 14.0 |  | 18 | 13.9 | Ref. |  |  |
|  | G | 258 | 86.0 |  | 112 | 86.2 | 1.05 | (0.57, 1.93) | 0.88 |
| rs112779426 | GG | 151 | 100.0 |  | 65 | 100.0 |  |  |  |
| exon | GA | 0 | 0.0 |  | 0 | 0.0 |  |  |  |
|  | AA | 0 | 0.0 |  | 0 | 0.0 |  |  |  |
|  | G | 302 | 100.0 |  | 130 | 100.0 |  |  |  |
|  | A | 0 | 0.0 |  | 0 | 0.0 |  |  |  |
| rs146560583 | GG | 151 | 100.0 |  | 65 | 100.0 |  |  |  |
| exon | GA | 0 | 0.0 |  | 0 | 0.0 |  |  |  |
|  | AA | 0 | 0.0 |  | 0 | 0.0 |  |  |  |
|  | G | 302 | 100.0 |  | 130 | 100.0 |  |  |  |
|  | A | 0 | 0.0 |  | 0 | 0.0 |  |  |  |
| rs143655258 | TT | 151 | 100.0 |  | 65 | 100.0 |  |  |  |
| exon | TC | 0 | 0.0 |  | 0 | 0.0 |  |  |  |
|  | CC | 0 | 0.0 |  | 0 | 0.0 |  |  |  |
|  | T | 302 | 100.0 |  | 130 | 100.0 |  |  |  |
|  | C | 0 | 0.0 |  | 0 | 0.0 |  |  |  |
| rs147159813 | CC | 151 | 100.0 |  | 65 | 100.0 |  |  |  |
| exon | CT | 0 | 0.0 |  | 0 | 0.0 |  |  |  |
|  | TT | 0 | 0.0 |  | 0 | 0.0 |  |  |  |
|  | C | 302 | 100.0 |  | 130 | 100.0 |  |  |  |
|  | T | 0 | 0.0 |  | 0 | 0.0 |  |  |  |
| rs117600063 | TT | 149 | 98.0 |  | 65 | 100.0 | Ref. |  |  |
| exon | TA | 10 | 2.0 |  | 0 | 0.0 | - |  |  |
|  | AA | 0 | 0.0 |  | 0 | 0.0 | - |  |  |
|  | T | 299 | 99.0 |  | 130 | 100.0 | Ref. |  |  |
|  | A | 3 | 1.0 |  | 0 | 0.0 | - |  |  |
| rs7676834 | GG | 27 | 17.9 |  | 6 | 9.2 | Ref. |  |  |
| intron | CG | 72 | 47.7 |  | 31 | 47.7 | 2.03 | (0.75, 5.52) | 0.16 |
|  | CC | 52 | 34.4 |  | 28 | 43.1 | 2.35 | (0.85, 6.49) | 0.10 |
|  | G | 126 | 41.7 |  | 43 | 33.1 | Ref. |  |  |
|  | C | 176 | 58.3 |  | 87 | 66.9 | 1.41 | (0.91, 2.19) | 0.13 |
| rs73826386 | CC | 151 | 100.0 |  | 65 | 100.0 |  |  |  |
| exon | CT | 0 | 0.0 |  | 0 | 0.0 |  |  |  |
|  | TT | 0 | 0.0 |  | 0 | 0.0 |  |  |  |
|  | C | 302 | 100.0 |  | 130 | 100.0 |  |  |  |
|  | T | 0 | 0.0 |  | 0 | 0.0 |  |  |  |
| rs17001594 | AA | 35 | 23.3 |  | 8 | 12.3 | Ref. |  |  |
| intron | GA | 78 | 52.0 |  | 34 | 52.3 | 1.86 | (0.77, 4.49) | 0.17 |
|  | GG | 37 | 24.7 |  | 23 | 35.4 | 2.60 | (1.01, 6.69) | 0.05 |
|  | A | 148 | 49.3 |  | 50 | 38.5 | Ref. |  |  |
|  | G | 152 | 50.7 |  | 80 | 61.5 | 1.53 | (0.99, 2.35) | 0.05 |
| rs6824953 | GG | 78 | 52.0 |  | 29 | 44.6 | Ref. |  |  |
| intron | GC | 58 | 38.7 |  | 28 | 43.1 | 1.32 | (0.70, 2.50) | 0.39 |
|  | CC | 14 | 9.3 |  | 8 | 12.3 | 1.72 | (0.63, 4.75) | 0.29 |
|  | G | 214 | 71.3 |  | 86 | 66.2 | Ref. |  |  |
|  | C | 86 | 28.7 |  | 44 | 33.9 | 1.33 | (0.84, 2.10) | 0.22 |
| rs6825004 | CC | 71 | 47.3 |  | 25 | 38.5 | Ref. |  |  |
| intron | CG | 67 | 44.7 |  | 33 | 50.8 | 1.40 | (0.74, 2.64) | 0.30 |
|  | GG | 12 | 8.0 |  | 7 | 10.8 | 2.11 | (0.70, 6.32) | 0.18 |
|  | C | 209 | 69.7 |  | 83 | 63.9 | Ref. |  |  |
|  | G | 91 | 30.3 |  | 47 | 36.2 | 1.38 | (0.88, 2.17) | 0.16 |
| rs17001606 | AA | 129 | 86.0 |  | 58 | 89.2 | 1.37 | (0.54, 3.45) | 0.51 |
| intron | AT | 21 | 14.0 |  | 7 | 10.8 | Ref. |  |  |
|  | TT | 0 | 0.0 |  | 0 | 0.0 | - |  |  |
|  | A | 279 | 93.0 |  | 123 | 94.6 | 1.34 | (0.55, 3.28) | 0.52 |
|  | T | 21 | 7.0 |  | 7 | 5.4 | Ref. |  |  |
| rs9994218 | TT | 144 | 95.4 |  | 59 | 90.8 | Ref. |  |  |
| intron | TC | 7 | 4.6 |  | 6 | 9.2 | 1.51 | (0.48, 4.78) | 0.49 |
|  | CC | 0 | 0.0 |  | 0 | 0.0 | - |  |  |
|  | T | 295 | 97.7 |  | 124 | 95.4 | Ref. |  |  |
|  | C | 7 | 2.3 |  | 6 | 4.6 | 1.48 | (0.48, 4.55) | 0.50 |
| rs11097262 | CC | 77 | 51.3 |  | 30 | 46.2 | Ref. |  |  |
| intron | CT | 62 | 41.3 |  | 28 | 43.1 | 1.17 | (0.62, 2.20) | 0.63 |
|  | TT | 11 | 7.3 |  | 7 | 10.8 | 2.16 | (0.71, 6.54) | 0.17 |
|  | C | 216 | 72.0 |  | 88 | 67.7 | Ref. |  |  |
|  | T | 84 | 28.0 |  | 42 | 32.3 | 1.32 | (0.83, 2.09) | 0.24 |
| rs6852859 | GG | 50 | 33.1 |  | 28 | 43.1 | 2.45 | (0.89, 6.77) | 0.08 |
| intron | GA | 74 | 49.0 |  | 31 | 47.7 | 1.97 | (0.73, 5.34) | 0.18 |
|  | AA | 27 | 17.9 |  | 6 | 9.2 | Ref. |  |  |
|  | G | 174 | 57.6 |  | 87 | 66.9 | 1.45 | (0.94, 2.26) | 0.10 |
|  | A | 128 | 42.4 |  | 43 | 33.1 | Ref. |  |  |
| rs1542093 | AA | 130 | 86.1 |  | 58 | 89.2 | 1.36 | (0.54, 3.43) | 0.52 |
| intron | AG | 21 | 13.9 |  | 7 | 10.8 | Ref. |  |  |
|  | GG | 0 | 0.0 |  | 0 | 0.0 | - |  |  |
|  | A | 281 | 93.1 |  | 123 | 94.6 | 1.33 | (0.54, 3.26) | 0.53 |
|  | G | 21 | 7.0 |  | 7 | 5.4 | Ref. |  |  |
| rs999361 | GG | 60 | 40.0 |  | 19 | 29.2 | Ref. |  |  |
| intron | GT | 69 | 46.0 |  | 37 | 56.9 | 1.69 | (0.87, 3.30) | 0.12 |
|  | TT | 21 | 14.0 |  | 9 | 13.9 | 1.41 | (0.54, 3.68) | 0.48 |
|  | G | 189 | 63.0 |  | 75 | 57.7 | Ref. |  |  |
|  | T | 111 | 37.0 |  | 55 | 42.3 | 1.27 | (0.82, 1.95) | 0.28 |
| rs17001622 | CC | 51 | 34.0 |  | 28 | 43.1 | 2.29 | (0.83, 6.34) | 0.11 |
| intron | CA | 73 | 48.7 |  | 31 | 47.7 | 1.86 | (0.69, 5.06) | 0.22 |
|  | AA | 26 | 17.3 |  | 6 | 9.2 | Ref. |  |  |
|  | C | 175 | 58.3 |  | 87 | 66.9 | 1.41 | (0.91, 2.19) | 0.13 |
|  | A | 125 | 41.7 |  | 43 | 33.1 | Ref. |  |  |
| rs17001640 | AA | 43 | 28.5 |  | 23 | 35.4 | 1.74 | (0.73, 4.12) | 0.21 |
| intron | AG | 73 | 48.3 |  | 31 | 47.7 | 1.56 | (0.69, 3.54) | 0.29 |
|  | GG | 35 | 23.2 |  | 11 | 16.9 | Ref. |  |  |
|  | A | 159 | 52.6 |  | 77 | 59.2 | 1.31 | (0.85, 2.00) | 0.22 |
|  | G | 143 | 47.4 |  | 53 | 40.8 | Ref. |  |  |
| rs12508946 | TT | 126 | 84.0 |  | 54 | 83.1 | 0.87 | (0.39, 1.96) | 0.74 |
| intron | TA | 21 | 14.0 |  | 11 | 16.9 | Ref. |  |  |
|  | AA | 3 | 2.0 |  | 0 | 0.0 | - |  |  |
|  | T | 273 | 91.0 |  | 119 | 91.5 | 1.13 | (0.54, 2.39) | 0.75 |
|  | A | 27 | 9.0 |  | 11 | 8.5 | Ref. |  |  |
| rs17001659 | TT | 64 | 42.7 |  | 31 | 47.7 | 2.58 | (0.67, 8.90) | 0.17 |
| intron | TC | 72 | 48.0 |  | 31 | 47.7 | 2.08 | (0.55, 7.93) | 0.28 |
|  | CC | 14 | 9.3 |  | 3 | 4.6 | Ref. |  |  |
|  | T | 200 | 66.7 |  | 93 | 71.5 | 1.35 | (0.85, 2.14) | 0.21 |
|  | C | 100 | 33.3 |  | 37 | 28.5 | Ref. |  |  |
| rs12640238 | TT | 64 | 42.4 |  | 31 | 47.7 | 1.90 | (0.56, 6.44) | 0.30 |
| intron | TG | 73 | 48.3 |  | 30 | 46.2 | 1.46 | (0.44, 4.91) | 0.54 |
|  | GG | 14 | 9.3 |  | 4 | 6.2 | Ref. |  |  |
|  | T | 201 | 66.6 |  | 92 | 70.8 | 1.30 | (0.82, 2.06) | 0.26 |
|  | G | 101 | 33.4 |  | 38 | 29.2 | Ref. |  |  |
| **ANXA2** |  |  |  |  |  |  |  |  |  |
| rs7163836 | TT | 43 | 28.9 |  | 23 | 35.4 | 1.56 | (0.66, 3.70) | 0.31 |
| intron | CT | 76 | 51.0 |  | 30 | 46.2 | 1.13 | (0.50, 2.55) | 0.76 |
|  | CC | 30 | 20.1 |  | 12 | 18.5 | Ref. |  |  |
|  | T | 162 | 54.4 |  | 76 | 58.5 | 1.27 | (0.83, 1.94) | 0.28 |
|  | C | 136 | 45.6 |  | 54 | 41.5 | Ref. |  |  |
| rs11854079 | AA | 63 | 42.3 |  | 32 | 49.2 | 1.83 | (0.72, 4.67) | 0.21 |
| intron | AG | 61 | 40.9 |  | 25 | 38.5 | 1.51 | (0.58, 3.95) | 0.40 |
|  | GG | 25 | 16.8 |  | 8 | 12.3 | Ref. |  |  |
|  | A | 187 | 62.8 |  | 89 | 68.5 | 1.36 | (0.87, 2.13) | 0.18 |
|  | G | 111 | 37.3 |  | 41 | 31.5 | Ref. |  |  |
| rs11629852 | TT | 104 | 69.8 |  | 41 | 63.1 | Ref. |  |  |
| intron | TG | 36 | 24.2 |  | 19 | 29.2 | 1.41 | (0.72, 2.81) | 0.32 |
|  | GG | 9 | 6.0 |  | 5 | 7.7 | 1.53 | (0.47, 5.01) | 0.48 |
|  | T | 244 | 81.9 |  | 101 | 77.7 | Ref. |  |  |
|  | G | 54 | 18.1 |  | 29 | 22.3 | 1.37 | (0.81, 2.31) | 0.24 |
| rs11071521 | GG | 62 | 41.3 |  | 33 | 50.8 | 2.10 | (0.83, 5.28) | 0.12 |
| intron | GT | 60 | 40.0 |  | 24 | 36.9 | 1.55 | (0.60, 4.01) | 0.37 |
|  | TT | 28 | 18.7 |  | 8 | 12.3 | Ref. |  |  |
|  | G | 184 | 61.3 |  | 90 | 69.2 | 1.50 | (0.95, 2.35) | 0.08 |
|  | T | 116 | 38.7 |  | 40 | 30.8 | Ref. |  |  |
| rs8030787 | CC | 53 | 35.6 |  | 21 | 32.3 | Ref. |  |  |
| intron | CT | 69 | 46.3 |  | 30 | 46.2 | 1.10 | (0.56, 2.18) | 0.78 |
|  | TT | 27 | 18.1 |  | 14 | 21.5 | 1.26 | (0.54, 2.92) | 0.60 |
|  | C | 175 | 58.7 |  | 72 | 55.4 | Ref. |  |  |
|  | T | 123 | 41.3 |  | 58 | 44.6 | 1.12 | (0.73, 1.72) | 0.59 |
| rs1551347 | AA | 70 | 47.0 |  | 34 | 52.3 | 1.83 | (0.46, 7.29) | 0.39 |
| intron | AG | 68 | 45.6 |  | 28 | 43.1 | 1.58 | (0.39, 6.33) | 0.52 |
|  | GG | 11 | 7.4 |  | 3 | 4.6 | Ref. |  |  |
|  | A | 208 | 69.8 |  | 96 | 73.9 | 1.22 | (0.76, 1.96) | 0.42 |
|  | G | 90 | 30.2 |  | 34 | 26.2 | Ref. |  |  |
| rs4775262 | CC | 92 | 61.7 |  | 47 | 72.3 | 1.80 | (0.32, 10.00) | 0.50 |
| intron | CT | 52 | 34.9 |  | 16 | 24.6 | 1.07 | (0.18, 6.34) | 0.94 |
|  | TT | 5 | 3.4 |  | 2 | 3.1 | Ref. |  |  |
|  | C | 236 | 79.2 |  | 110 | 84.6 | 1.55 | (0.88, 2.72) | 0.13 |
|  | T | 62 | 20.8 |  | 20 | 15.4 | Ref. |  |  |
| rs9920823 | GG | 98 | 65.3 |  | 39 | 60.0 | Ref. |  |  |
| intron | GT | 46 | 30.7 |  | 22 | 33.9 | 1.09 | (0.57, 2.09) | 0.79 |
|  | TT | 6 | 4.0 |  | 4 | 6.2 | 1.28 | (0.33, 4.92) | 0.72 |
|  | G | 242 | 80.7 |  | 100 | 76.9 | Ref. |  |  |
|  | T | 58 | 19.3 |  | 30 | 23.1 | 1.11 | (0.67, 1.86) | 0.68 |
| rs17237276 | AA | 1 | 0.7 |  | 0 | 0.0 | - |  |  |
| intron | GA | 15 | 10.0 |  | 6 | 9.2 | Ref. |  |  |
|  | GG | 134 | 89.3 |  | 59 | 90.8 | 1.03 | (0.37, 2.90) | 0.95 |
|  | A | 17 | 5.7 |  | 6 | 4.6 | Ref. |  |  |
|  | G | 283 | 94.3 |  | 124 | 95.4 | 1.12 | (0.42, 3.01) | 0.82 |
| rs16942555 | GG | 1 | 0.7 |  | 0 | 0.0 | - |  |  |
| intron | TG | 24 | 16.1 |  | 9 | 13.9 | Ref. |  |  |
|  | TT | 124 | 83.2 |  | 56 | 86.2 | 1.44 | (0.61, 3.39) | 0.41 |
|  | G | 26 | 8.7 |  | 9 | 6.9 | Ref. |  |  |
|  | T | 272 | 91.3 |  | 121 | 93.1 | 1.50 | (0.67, 3.35) | 0.33 |
| rs8033800 | TT | 5 | 3.3 |  | 2 | 3.1 | Ref. |  |  |
| intron | AT | 45 | 29.8 |  | 19 | 29.2 | 0.80 | (0.14, 4.66) | 0.81 |
|  | AA | 101 | 66.9 |  | 44 | 67.7 | 0.93 | (0.17, 5.15) | 0.94 |
|  | T | 55 | 18.2 |  | 23 | 17.7 | Ref. |  |  |
|  | A | 247 | 81.8 |  | 107 | 82.3 | 1.09 | (0.63, 1.89) | 0.77 |
| **PSGL-1 (SELPLG)** |  |  |  |  |  |  |  |  |  |
| rs2228315 | AA | 22 | 14.7 |  | 4 | 6.2 | Ref. |  |  |
| exon | GA | 63 | 42.0 |  | 29 | 44.6 | 2.48 | (0.76, 8.06) | 0.13 |
|  | GG | 65 | 43.3 |  | 32 | 49.2 | 2.67 | (0.83, 8.58) | 0.10 |
|  | A | 107 | 35.7 |  | 37 | 28.5 | Ref. |  |  |
|  | G | 193 | 64.3 |  | 93 | 71.5 | 1.39 | (0.88, 2.21) | 0.16 |
| rs7137098 | TT | 63 | 41.7 |  | 20 | 30.8 | Ref. |  |  |
| intron | TA | 74 | 49.0 |  | 30 | 46.2 | 1.24 | (0.63, 2.44) | 0.53 |
|  | AA | 14 | 9.3 |  | 15 | 23.1 | 2.75 | (1.11, 6.81) | 0.03 |
|  | T | 200 | 66.2 |  | 70 | 53.9 | Ref. |  |  |
|  | A | 102 | 33.8 |  | 60 | 46.2 | 1.55 | (1.01, 2.39) | 0.04 |
| rs8179137 | AA | 96 | 63.6 |  | 32 | 49.2 | Ref. |  |  |
| intron | AG | 52 | 34.4 |  | 27 | 41.5 | 1.58 | (0.84, 2.99) | 0.16 |
|  | GG | 3 | 2.0 |  | 6 | 9.2 | 5.27 | (1.17, 23.69) | 0.03 |
|  | A | 244 | 80.8 |  | 91 | 70.0 | Ref. |  |  |
|  | G | 58 | 19.2 |  | 39 | 30.0 | 1.77 | (1.09, 2.88) | 0.02 |
| rs3782522 | AA | 27 | 18.0 |  | 12 | 18.5 | Ref. |  |  |
| intron | GA | 78 | 52.0 |  | 33 | 50.8 | 1.12 | (0.49, 2.55) | 0.78 |
|  | GG | 45 | 30.0 |  | 20 | 30.8 | 1.17 | (0.48, 2.85) | 0.73 |
|  | A | 132 | 44.0 |  | 57 | 43.9 | Ref. |  |  |
|  | G | 168 | 56.0 |  | 73 | 56.2 | 1.07 | (0.70, 1.65) | 0.75 |
| rs3782520 | GG | 141 | 94.6 |  | 58 | 89.2 | Ref. |  |  |
| intron | GA | 8 | 5.4 |  | 6 | 9.2 | 1.74 | (0.55, 5.52) | 0.35 |
|  | AA | 0 | 0.0 |  | 1 | 1.5 | - |  |  |
|  | G | 290 | 97.3 |  | 122 | 93.9 | Ref. |  |  |
|  | A | 8 | 2.7 |  | 8 | 6.2 | 2.22 | (0.77, 6.39) | 0.14 |

EV71, enterovirus 71; SCARB2, scavenger receptor class B member 2; PSGL-1, P-selectin glycoprotein ligand-1; ANXA2, annexin II.

^a^The mild group including uncomplicated EV71 infection cases, or myoclonic jerk, or aseptic meningitis.

^b^The severe group including severe EV71 infection cases of encephalitis, polio-like syndrome or encephalomyelitis with or without cardiopulmonary failure.

^c^The ORs, 95% CI, and P values were calculated by multivariate logistic regression with adjustment of age and gender.
